# Lectures on Diseases of the Skin

**Published:** 1887-03

**Authors:** Henry Wile

**Affiliations:** Lecturer on Dermatology, Atlanta, Ga.


THE ATLANTA MEDICAL AND SURGICAL JOURNAL.

VOL. IV. MARCH, 1887. NO. 1.

### Original Communications.

### LECTURES ON DISEASES OF THE SKIN.

DELIVERED AT THE ATLANTA MEDICAL COLLEGE DURING THE SESSION OF 1885-86, BY HENRY WILE, M. D., LECTURER ON DERMATOLOGY, ATLANTA, GA.

### LECTURE XIII.

### ECZEMA CONCLUDED—EXTERNAL TREATMENT—HERPES FACIALIS—HERPES PROGENITALIS.

Reported Stenographically for THE ATLANTA MEDICAL AND SURGICAL JOURNAL by W. Kay Tewksbury.

The external treatment of eczema is always in order, and in every case it is of the first importance to determine whether the process is acute or chronic. In the management of acute forms of eczema, as before said, remedies of a soothing, protecting, non-irritating character are indicated. For instance, in the acute vesicular variety the process may often be brought to a speedy and favorable termination by employing the method recommended by Duhring. This consists of the application of black wash composed of calomel, about a drachm to six or eight ounces of lime water, three or four times a day, several minutes at a time, followed by the use of oxide of zinc ointment spread on muslin rags. The black wash may be substituted by the following:

℞. Pulveris calamini.

Zinci oxidi . . . . . . . . . . . . . . . . . . . . . . . . aa ʒii.

Glycerini . . . . . . . . . . . . . . . . . . . . . . . . . . . fʒii.

Aquæ rosæ . . . . . . . . . . . . . . . . . . . . . . . . f℥iv.

M. Sig. Apply several times daily. Shake bottle.

If the discharge is very profuse some absorbent powder should be used, it being frequently dusted over the part. For this purpose oxide of zinc, subnitrate of bismuth, carbonate of magnesia, Venetian talc, finely powdered starch, are available. One of the best of these is Venetian talc, a fine and impalpable powder, which can be used in combination with oxide of zinc and starch, equal parts of each. The frequent use of the powder is especially indicated when the disease attacks parts of the skin that are in apposition. In that event it is even advisable to place, in addition to the powder, pledgets of absorbent cotton, renewed at short intervals, between the folds of skin.

In acute papular eczema the itching is usually more intense than in any other variety, and to ease this troublesome symptom carbolic acid is a reliable remedy, used in the proportion of from one to three drachms to a pint of water, sponging the parts several times a day. The application of water as hot as can be borne, alcohol or whiskey, pure or diluted, or a saturated solution of boracic acid, are also agents of decided value in this respect. When the acute symptoms in acute vesicular eczema have subsided in a measure, when the discharge is diminished and there is a tendency to desquamation, the remedies may be made slightly stimulating. Thus the lotion may be discontinued and calomel, ten to twenty grains, may be added to the ointment of oxide of zinc, together with five to ten drops of carbolic acid to control itching. Instead of calomel, ammoniated mercury can be used. Likewise salicylic acid in the form of a paste as follows:

℞. Acidi salicylici........................℥ss.

Acidi carbolici........................gttv.

Amyli..............................

Zinci oxidi.........................aa ℥ss.

Ung. petrolati........................℥vii.

M. ft. ungt.

Sig. Spread on muslin rag and apply.

If these slightly stimulating remedies seem to disagree with the lesions by aggravating the symptoms, they should be discontinued at once and the milder ones resumed. Should, however, the desquamation continue, the above remedies may be made more stimulating until desquamation ceases and the skin returns to its normal condition.

In chronic eczema it is first necessary to remove any crusts, accumulations of scales or other matter that would keep the remedy from coming in contact with the diseased surface. For this purpose solvents are employed. When these accumulations are scanty a few applications of hot water, with glycerine, castile or salicylic acid soap, or hot poultices, will suffice. When, however, they are abundant and adherent, as is often the case in chronic eczema of the scalp, they are best removed by first making applications of olive or almond oil, lard or vaseline. Any one of these may be selected, and the parts are to be thoroughly anointed and covered with a flannel rag. After several hours, if the crusts are not softened, another application must be made. When the crusts are soft and friable they are to be washed off with hot water and soap. Should the exposed surface be intensely hyperæmic and oozing, mild remedies, such as oxide of zinc ointment, calomel ten to twenty grains, carbolic acid five drops to one ounce of oxide of zinc ointment may be used and in general the treatment for the acute process may be carried out. Stimulation is desirable, and irritating agents are at times necessary to produce an acute state, or to bring about the substitution of an acute process for a chronic one, with the idea of having to deal with a condition in which the changes are going on rapidly and a *restitutio ad integrum* may be more easily effected. Thus, in circumscribed spots that present infiltration and thickening, the application twice daily of sapo viridis is usually attended with good results. It must be used persistently and only suspended in case of marked irritation. A mild ointment is then applied, and as soon as acute symptoms subside the soap application may be resumed and thus continued until the thickening disappears.

In chronic eczema of the squamous variety, where there is much hyperæmia and little thickening, and in those cases where there is no oozing after a removal of the crusts, the use of tar in some form is expedient. Oleum cadinum (oil of juniper) is recommended on account of its thinness when compared with pix liquida (oil of pine). It may be used in the form of an ointment, one-half to two drachms to the ounce of vaseline or lard, or as a lotion dissolved in equal parts of alcohol or olive oil. The strength of the preparation will always depend upon the amount of stimulation required. As an ointment it must be thoroughly rubbed into the lesions for five or ten minutes at a time by means of a flannel rag, and as a lotion it should be painted on with a coarse brush. An eligible preparation is that first proposed by Bulkley, and known as liquor picis alkalinus. It contains two drachms of pix liquida, one drachm of caustic potash and five drachms of water. In the form of an ointment or lotion, it is used in the proportion of one to four drachms to the ounce. It is especially indicated where there is infiltration and marked thickening of the epidermis, the potassa having a solvent effect upon the cellular layers of the epidermis. This preparation must be used with caution on account of the action of the potassa. Another efficient combination for this purpose is as follows:

℞. Olei cadini............................f℥iss.

Glycerini..............................fʒss.

Spts. saponat. kalin......................f℥iv.

M.

Sig. Apply once or twice daily.

In the palms of the hands the application of pure carbolic acid is often attended with the best results. In a short time the thickening disappears. It remains to be said of all tarry preparations that some individuals are peculiarly sensitive to tar, and when applications are made over large surfaces it may be absorbed and give rise to toxic symptoms (gastric disturbances, black vomit, dark urine). Locally it may prove irritating to the sebaceous glands, causing an eruption of *tar acne*. Then again, it may prove irritating to the lesions themselves instead of reducing the hyperæmia, promoting absorption and stopping the desquamation. For these reasons it is always better to apply the agent first over a small surface and see the effect. When applied to surfaces that are in apposition, care must be taken to keep such surfaces apart by means of absorbent cotton.

The location of the disease in many instances determines the method of treatment and selection of remedies. On the scalp, as a rule, stronger remedies may be applied than on any other part of the body. It must be borne in mind that all accumulations of scale and crust must be removed by means of solvent oils or soaps, so that the medication may be effective; then the application of the ointment is made two or three times a day, well rubbed into the affected part. When the process is confined to the occipital region, it will, in the vast majority of cases, be found associated with pediculosis. The head should then be thoroughly anointed with petroleum and covered with a flannel cap. After twelve hours the parts should be washed with castile soap and hot water. The process is repeated according to the necessities of the case. Later, measures of cleanliness will usually suffice to bring about a cure. In the squamous variety, tar acts well and can be used in strong proportions, as one to two drachms of the oil of cade to one ounce of vaseline. It may prove advantageous to combine with the tar precipitated sulphur—one to four drachms to the ounce of vaseline. In pustular eczema of the scalp ointments of calomel and ammoniated mercury and salicylic acid are recommended.

In erythematous eczema of the face, especially of the forehead, saturated solution of boracic acid, or sulphate of zinc one to two drachms to a pint of water applied several times daily for several minutes at a time will often give marked relief, especially from the burning, itching symptoms, and reduce the hyperæmia. An excellent formula is as follows: Sulphate of zinc, sulphuret of potash, each one-half to one drachm, glycerine two drachms to four ounces of rose water. This is to be used as the other lotions. This form often proves obstinate and passes into a squamous condition in which the tarry preparations may be employed with good results.

In eczema of the lips a common form is that encountered on the upper lip, involving the end of the nose or alæ and usually due to catarrhal discharges from the nostrils. The object in this case is first to remove the cause which consists in the treatment of the nasal catarrh ; the irritating discharges running down upon the lip produce excoriation, resulting in eczema. After the discharge has been arrested by the injection of hot water and astringents, especially solutions of nitrate of silver, the disease of the skin responds readily to stimulating remedies as already indicated. Sometimes the application of simple oxide of zinc ointment will bring about favorable results. For chaps or fissures oxide of zinc ointment or vaseline, pure or combined with a few drops of carbolic acid or cold cream, is generally all that is required. The remedy should be applied frequently, and especially when the parts are to be exposed. In obstinate cases of fissure of the lips and nostrils a few applications of sapo viridis, the use of nitrate of silver stick or a strong solution of potash, followed by soothing application, is indicated.

Eczema of the nipple and areola or of the lower edge of the gland mammary, where it comes in apposition with the skin of the thorax, is not an uncommon affection and often presents itself in a chronic form with the development of painful fissures. Where there is moisture or oozing the treatment is the same as already indicated for acute cases. Where there is a tendency to desquamation, strong calomel ointments and the salycilic acid paste referred to above, or any of the tarry preparations, may be used with satisfaction.

Where there is a large development of the mammary glands, and especially in corpulent women who perspire freely, the skin of the gland, by being in constant apposition on its lower border with the skin of the thorax, may become the seat of chronic eczema rubrum. The treatment may consist of bathing the parts occasionally during the day with a saturated solution of boracic acid or weak solutions of alum or tannic acid. In the intervals between the liquid applications the skin must not be allowed to come in apposition, but the folds should be kept apart by means of absorbent cotton well dusted with powder. When the squamous form is reached it will be necessary to use more stimulating applications, such as liquor picis alkalines one drachm to the ounce of water. Should the fissures of the nipple prove obstinate the same treatment can be resorted to as is recommended in cases of obstinate fissure of the lips.

In discussing the pathology of this disease a form known as Paget's disease of the nipple was referred to, in which a malignant process involving the structure of the gland was developed. Cases where this extreme and malignant condition develops are rare, but several have been reported and should be borne in mind when the disease is of long standing and where the nipple has become retracted. The question of excision of the gland should then be considered.

Eczema occurring about the genitalia, especially on the scrotum, the perineum and around the anus, is common, chronic, rebellious to treatment. The itching in these forms is a prominent symptom and always distressing to the patient. It is also attended with thickening of the skin. In these cases, after all oozing has been checked, the tarry preparations are invaluable as a relief to the itching and to reduce hyperæmia. If the scrotum is affected it should be carried in a suspensory bandage, and the neighboring parts are to be kept dry by frequent use of some dusting powder.

Eczema fissum occurring upon the fingers and backs of the hands, especially about the joints, and making its appearance in cold weather, is satisfactorily treated by the use of rubber gloves or kid gloves, such parts of them being used as are required. The patient should be directed to wear the same continuously night and day, or as much as would be convenient. When the fissures are deep and painful the strong remedies already mentioned for these lesions are to be used.

For localized patches of squamous eczema, accompanied by great thickening of the epidermis, as occurs often on the palms and backs of the hands or soles and backs of the feet, a series of applications with sapo viridis, to be kept applied until the parts become irritated, will effectually reduce the thickening. Soothing agents are then used. One such treatment may not do the work entirely, so that after the irritation has subsided it may be necessary to repeat the procedure.

HERPES is an acute vesicular inflammation of the skin, appearing in two forms, named after the locality affected—*herpes facialis* and *herpes progenitalis*. *Herpes facialis* is characterized by the appearance of small clusters of vesicle which develop quickly and in a few days dry up, forming brownish crusts. Their development is attended with slight burning. The lesions appear particularly about the lips, even upon the mucous membrane, having there the appearance of superficial grayish abrasions, and the process, therefore, sometimes called *herpes labialis*. The eruption makes its appearance in the course of acute disease, especially at the termination of fevers. It is, therefore, known popularly as fever blister.

*Herpes progenitalis* presents some importance, in that it may be confounded with venereal disease. It occurs in the male upon the glans or body of the penis or the surface of the prepuce. It makes its appearance in the form of groups of vesicles, but situated on the inner surface of the prepuce, and the discharge being retained upon the glans penis it may give rise to irritation, resulting in balanitis. In the female it appears about the clitoris and labiora minora. The treatment for both forms is simple. Where it occurs upon the face astringent ointments, as oxide of zinc, will suffice. Where it exists upon the glans penis or prepuce it is necessary to keep the parts dry by the use of absorbent powders and pledgets of cotton, renewed frequently during the day. These forms occurring on the female may receive the same treatment. The disease disappears under this treatment in the course of a few days.

